# MeteoCoast-EC: An open-access hourly meteorological dataset from automatic weather stations in coastal Ecuador

**DOI:** 10.1016/j.dib.2026.113233

**Published:** 2026-09-08

**Authors:** Jorge Parraga-Alava, Edison Solorzano, Emilio Jarre-Castro, Jose Zambrano, Ezequiel Zamora-Ledezma

**Affiliations:** a Facultad de Ciencias Informáticas, Universidad Técnica de Manabí, 130103, Portoviejo, Ecuador; b Laboratorio Funcionamiento de Agroecosistemas y Cambio Climático (FAGROCLIM), Departamento de Ciencias Agrícolas, Facultad de Ingeniería Agrícola, Universidad Técnica de Manabí, 13132 Lodana, Ecuador

**Keywords:** Meteorological dataset, Automatic weather stations, Coastal Ecuador, ENSO, Tropical climatology, Open-access time series

## Abstract

MeteoCoast-EC is an open-access, multi-variable meteorological dataset comprising 62,815 hourly records collected between January 2022 and May 2026 from three automatic weather stations operated by the Universidad Técnica de Manabí (UTM) in the coastal region of Ecuador: Santa Ana, Portoviejo, and Calderón, located at 50, 64, and 150 m a.s.l. respectively. The dataset addresses a critical observational gap: the coastal region of Ecuador holds the lowest density of operational meteorological stations of any region in the country, and INAMHI (Instituto Nacional de Meteorología e Hidrología) does not currently maintain active stations at the Portoviejo and Calderón sites. This gap is particularly consequential given that the hydrographic systems encompassing these stations experienced extreme precipitation anomalies affecting approximately 93% of basin areas during the 1997-1998 El Niño event, and extreme El Niño events are projected to increase in frequency under ongoing climate change. The monitored area also constitutes the primary agricultural and artisanal fishing zone of Ecuador, where locally observed, sub-daily meteorological records are essential for agroclimatic risk modeling, hydrological forecasting, and early warning system development. Two files are released. The raw export preserves the 75 columns delivered by the WeatherLink API together with the SQL export layer, 68 API fields plus 7 export fields, with no variable withheld. The processed release retains 23 analytically distinct variables, 4 identifiers and 19 measurements, and materializes them as 33 physical columns by adding 9 SI unit conversions and 1 auxiliary grouping key. Data are acquired using Davis Vantage Pro 2 sensors via a JavaScript-based ETL pipeline and stored in a PostgreSQL database at hourly resolution. Neither file is quality-controlled beyond deduplication, so the native sensor values are preserved. MeteoCoast-EC is released under CC BY 4.0 and deposited in Mendeley Data together with the complete processing and figure-generation code.

Specifications Table

**Table:** 

Subject: Earth and Planetary Sciences	Earth & Environmental Sciences
Specific subject area	*Tropical meteorology and climatology: ground-based atmospheric monitoring in coastal Ecuador for agroclimatic and ENSO research*
Type of data	Raw and processed. Two CSV files: MeteoCoast-EC_raw.csv, the complete 75-column raw export, and MeteoCoast-EC_processed.csv, the 33-column processed release, deposited together with the data dictionary, the station metadata, and the full processing and figure-generation code
Data collection	Sensor readings are retrieved every 60 seconds by an automated polling service from the WeatherLink v2 cloud API and stored as JSONB documents in a PostgreSQL 14 database. The export layer flattens them into CSV; the architecture supports alternative formats such as JSON or plain text as required
Data source location	*Country: Ecuador. Province: Manabí. Station coordinates (WGS84):* *Santa Ana (1.21°S, 80.36°W, 50 m a.s.l.);* *Portoviejo (1.05°S, 80.45°W, 64 m a.s.l.);* *Calderón (0.97°S, 80.36°W, 150 m a.s.l.)*
Data accessibility	Repository name: Mendeley Data Data identification number: https://www.doi.org/10.17632/bpsh7jtyd2.5 Direct URL to data: https://www.doi.org/10.17632/bpsh7jtyd2.5
Related research article	*None*

## 1 Value of the Data

• MeteoCoast-EC provides the only continuous, sub-daily, open-access meteorological records available at Portoviejo and Calderón, two locations within the Ecuadorian coastal network where INAMHI does not currently operate active stations [1]. The coastal region of Ecuador holds the lowest density of operational meteorological instruments relative to its territorial extent among all geographic regions of the country, making this dataset an irreplaceable resource for researchers, government agencies, and disaster risk management institutions that require ground-level observations for forecast calibration and early warning systems.
• Climatologists, hydrologists, and agronomists working on ENSO dynamics and extreme precipitation can use MeteoCoast-EC to validate and downscale model outputs and satellite-derived precipitation products such as the CHIRPS rainfall estimates [2] at the local level. The hydrographic basins of Portoviejo, Chone, and Jipijapa, encompassing the MeteoCoast-EC stations, have been identified as among the most severely affected by extreme El Niño events in Ecuador, with precipitation anomalies reaching extremely humid conditions (SPDI > 2.0) over approximately 93% of basin areas during the 1997–1998 event and sustained for up to ten consecutive months [3]. These basins are located within a region where the frequency of extreme El Niño events is projected to increase under ongoing greenhouse warming [4].
• Agricultural planners, agrometeorologists, and rural extension agencies operating in Manabí, the leading province in Ecuador for banana, cocoa, maize, and coffee production, can use the multi-year, multi-variable time series to develop site-specific agroclimatic indices, crop water requirement models, and seasonal risk assessments. The availability of solar radiation, UV index, and wind data alongside temperature and rainfall enables multi-variable crop and evapotranspiration modeling that is not feasible with standard single-variable station records, which is particularly relevant in a province where the agricultural sector is a major driver of economic growth [5].
• Data scientists and machine learning researchers can use MeteoCoast-EC as a benchmark dataset for time-series forecasting, anomaly detection, and transfer learning in under-observed tropical coastal environments, complementing existing high-volume meteorological datasets from other regions [6,7]. The multi-station architecture, 23-variables processed structure, and four-year temporal depth of the dataset provide the volume and heterogeneity required for training and evaluating deep learning models applicable to similar data-scarce regions in the Global South.
• Researchers studying the compound impacts of extreme El Niño events, hydrometeorological extremes, and air-quality crises in Latin America can integrate MeteoCoast-EC with existing analyses for Ecuador, Venezuela, and Brazil [3,8,9].
  Three reuse workflows illustrate how the release is intended to be consumed. A hydrologist can use the 15-minute peak rain rate and the daily accumulation for exploratory characterisation of rainfall events in the Portoviejo basin, accumulating a local record toward future intensity-duration-frequency analysis once the series is long enough to support defensible return-period estimation. An agrometeorologist can compute FAO Penman-Monteith reference evapotranspiration from temperature, humidity, solar radiation, and the 10-minute mean wind speed, and combine it with daily rainfall for a soil-water balance. A machine learning practitioner can frame 24-hour-ahead nowcasting as a multivariate sequence problem, using the window of concurrent operation as a spatial cross-validation split.

## 2 Background

The coastal region of Ecuador holds the lowest density of operational meteorological stations of any geographic region in the country, and the national meteorological agency (INAMHI) does not currently maintain active stations at the Portoviejo and Calderón sites. This observational gap motivated the deployment of the MeteoCoast-EC monitoring network by the Universidad Técnica de Manabí. The province of Manabí constitutes the primary agricultural and artisanal fishing zone of Ecuador, and the hydrographic basins encompassing the three stations have been documented as among those most severely affected by extreme El Niño events, which are projected to increase in frequency under ongoing greenhouse warming. These conditions establish a need for locally observed, sub-daily ground-based observations to support agroclimatic risk modeling, hydrological forecasting, and early warning system development in a data-scarce tropical coastal environment.

Methodologically, the three Davis Vantage Pro 2 stations were sited following WMO exposure guidelines along an approximately 80 km northwest-southeast transect that captures spatial variability across distinct microclimates. Readings are acquired through a JavaScript-based polling service that queries the WeatherLink v2 cloud API and stores records in a PostgreSQL database, with hourly aggregation and unit-conversion procedures designed to ensure reproducibility and downstream compatibility

## 3 Data Description

MeteoCoast-EC is distributed as two UTF-8 comma-separated values files. MeteoCoast-EC_raw.csv is the complete raw export: 75 columns and 1,171,323 records at the native polling cadence, of which 33,614 are duplicates under station_code and iso_date.[10] Here “raw” denotes an unfiltered database export rather than an unmodified sensor payload: 68 of the 75 columns arrive from the WeatherLink API and 7 are added by the export query, two of which, temp_min and temp_max, are derived daily extremes. MeteoCoast-EC_processed.csv is the hourly processed release: 33 columns and 62,815 rows, one per station and calendar hour, obtained by the aggregation of Section 4.4. Neither file is quality-controlled beyond deduplication. Sentinel codes and implausible readings are preserved as delivered by the sensor, and the filters of Section 4.6 are applied only inside the analysis script that produces the descriptive statistics and the two figures. Users requiring physically controlled data must run that pipeline or apply their own validated checks before any analysis that depends on physical plausibility.

The column arithmetic holds at every point in this article. The raw export is 68 API fields plus 7 fields added by the export query, that is 75 columns. Of these, 23 are retained and 52 are discarded, all 52 remaining available in the raw file. The 23 retained variables are 4 identifiers and 19 measurements; adding 9 SI conversions and the auxiliary key calendar_day gives 4 + 19 + 1 + 9 = 33 columns. Table 3 maps every raw column to its disposition, with subtotals. The uncompressed size is 611 MB for the raw export and 13 MB for the processed release.

### 3.1 Station overview

**Fig. 1:**
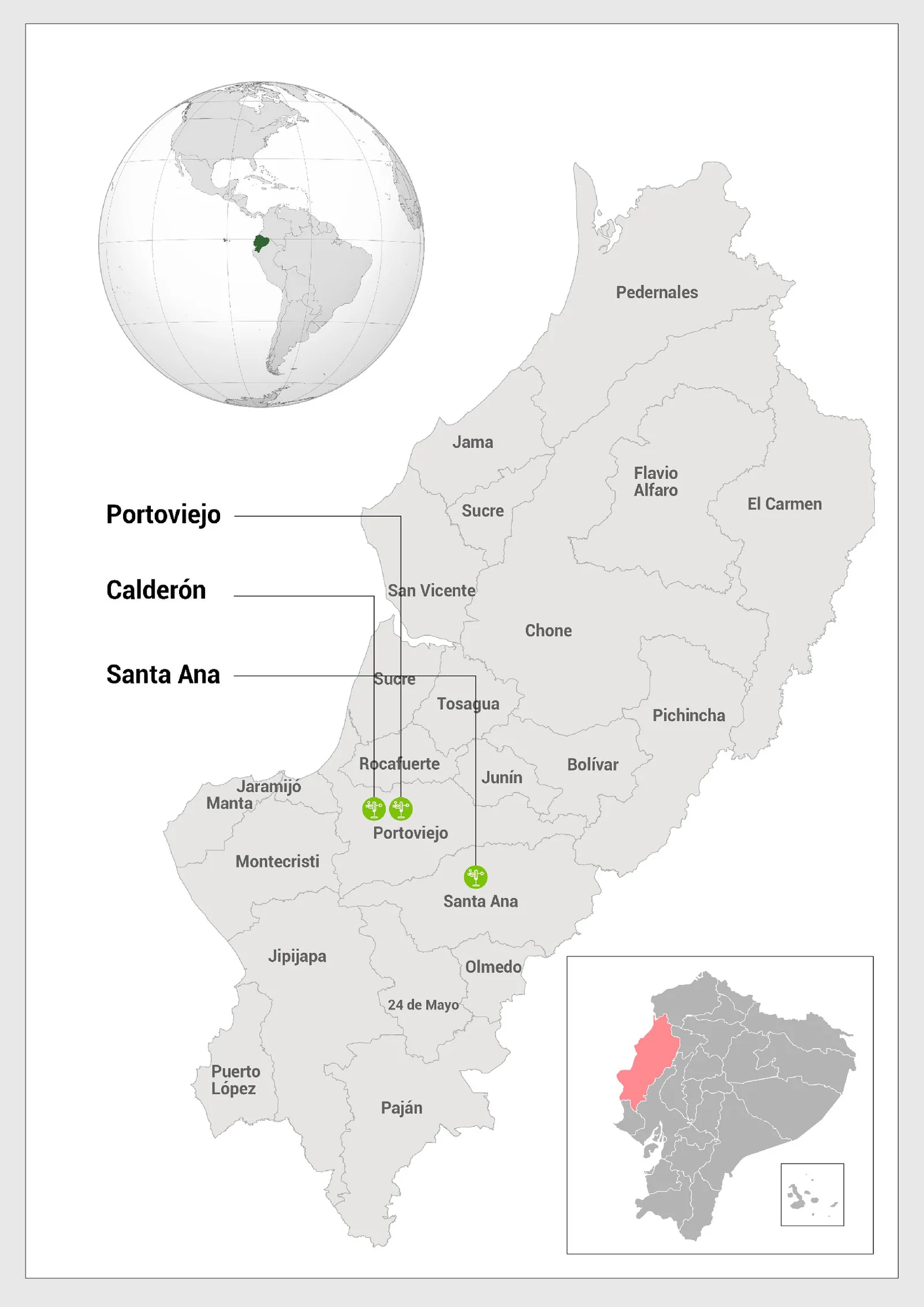
Geographic location of the three MeteoCoast-EC automatic weather stations in the coastal region of Ecuador.

Table 1 summarizes the three stations included in MeteoCoast-EC, including internal identifiers, geographic coordinates, temporal coverage, record count, and altitude above sea level. The record counts were recomputed directly on the released files for this revision and sum exactly to the total reported in the Abstract and in the opening of this section. The stations span a northwest-southeast transect of approximately 80 km across the coastal lowlands of Manabí province (Fig. 1). Similar multi-station monitoring approaches have been adopted in other regional networks in Latin America [6].

**Table 1:** Station metadata, temporal coverage, and recounted record totals. Coordinates in WGS84 decimal degrees. Altitude values estimated from the SRTM digital elevation model. The three record counts sum to 62,815.

Station name	Station ID	Lat (°S)	Lon (°W)	Alt (m a.s.l.)	Coverage start	Coverage end	Records (n)
Santa Ana	186565	-1.21	-80.36	50	2022-01-07	2026-05-16	30,384
Portoviejo	151434	-1.05	-80.45	64	2023-01-27	2026-05-16	27,453
Calderón	142673	-0.97	-80.36	150	2025-09-08	2026-05-16	4,978

Fig. 1 shows the geographic location of the three MeteoCoast-EC automatic weather stations deployed in the coastal region of Manabí province, Ecuador. The stations are plotted over a topographic base map with coordinates referenced in the WGS84 datum (decimal degrees) and altitude values derived from the SRTM digital elevation model. Santa Ana and Portoviejo are situated in low-elevation terrain toward the coast, while Calderón is located further inland at a higher elevation. The absence of active INAMHI reference stations at the Portoviejo and Calderón sites is indicated for reference.

### 3.2 Variable description

Table 2 provides a general overview of the structure and content of the fields delivered by the WeatherLink API, including the format, measurement representation, and semantic description of each recorded attribute. The dataset integrates multiple environmental and atmospheric observations collected through the monitoring infrastructure and API services described in this study. Sensor readings are stored using the original encoding and measurement conventions provided by the acquisition system to preserve consistency and reproducibility across data collection stages.

**Table 2:** Description of the 68 fields delivered by the WeatherLink API JSON payload. The 7 columns added by the SQL export layer are described in the text below, and Table 3 maps the resulting 75 raw columns to their disposition in the processed release.

N°	Column name	Type	Unit	Description
1	ts	INTEGER	-	Unix timestamp of the observation
2	hum	INTEGER	%	Relative humidity percentage
3	temp	FLOAT	°F	Instantaneous air temperature
4	tx_id	INTEGER	-	Transmitter identifier (Source ID)
5	rx_state	INTEGER	-	Receiver state or signal quality status
6	uv_index	FLOAT	-	Ultraviolet radiation index
7	wet_bulb	FLOAT	°F	Wet bulb temperature
8	dew_point	FLOAT	°F	Calculated dew point temperature
9	rain_size	INTEGER	-	Rainfall collector size or type identifier
10	solar_rad	FLOAT	W m⁻²	Solar radiation intensity
11	thw_index	FLOAT	°F	Temperature-Humidity-Wind perceived index
12	tz_offset	INTEGER	s	Timezone offset from UTC in seconds
13	heat_index	FLOAT	°F	Heat index based on temperature and humidity
14	thsw_index	FLOAT	°F	Temperature-Humidity-Sun-Wind index
15	wind_chill	FLOAT	°F	Calculated wind-chill factor
16	rain_storm_in	FLOAT	in	Rainfall during the current storm (inches)
17	rain_storm_mm	FLOAT	mm	Rainfall during the current storm (millimeters)
18	wind_dir_last	FLOAT	degrees	Last measured wind direction (0°–360°)
19	rain_rate_hi_in	FLOAT	in h⁻¹	Highest rain rate in current period (inches)
20	rain_rate_hi_mm	FLOAT	mm h⁻¹	Highest rain rate in current period (mm)
21	wind_speed_last	FLOAT	mph	Last measured instantaneous wind speed
22	rainfall_year_in	FLOAT	in	Cumulative yearly rainfall (inches)
23	rainfall_year_mm	FLOAT	mm	Cumulative yearly rainfall (millimeters)
24	rain_rate_last_in	FLOAT	in h⁻¹	Last measured rain rate (inches)
25	rain_rate_last_mm	FLOAT	mm h⁻¹	Last measured rain rate (millimeters)
26	rain_storm_clicks	INTEGER	-	Tipping bucket counts for the current storm
27	rainfall_daily_in	FLOAT	in	Daily cumulative rainfall (inches)
28	rainfall_daily_mm	FLOAT	mm	Daily cumulative rainfall (millimeters)
29	rain_storm_last_in	FLOAT	in	Total rainfall of the last storm (inches)
30	rain_storm_last_mm	FLOAT	mm	Total rainfall of the last storm (millimeters)
31	trans_battery_flag	INTEGER	-	Transmitter battery status (0 = Normal)
32	rain_rate_hi_clicks	INTEGER	-	Bucket counts for the highest rain rate
33	rainfall_monthly_in	FLOAT	in	Monthly cumulative rainfall (inches)
34	rainfall_monthly_mm	FLOAT	mm	Monthly cumulative rainfall (millimeters)
35	rainfall_year_clicks	INTEGER	-	Yearly tipping bucket cumulative counts
36	rain_rate_last_clicks	INTEGER	-	Tipping bucket counts for the last rain rate
37	rain_storm_start_time	INTEGER	-	Unix timestamp of current storm start
38	rainfall_daily_clicks	INTEGER	-	Daily tipping bucket cumulative counts
39	rain_storm_last_clicks	INTEGER	-	Bucket counts for the last storm event
40	rain_storm_last_end_at	INTEGER	-	Unix timestamp of the end of the last storm
41	rainfall_last_24_hr_in	FLOAT	in	Cumulative rainfall last 24 hours (inches)
42	rainfall_last_24_hr_mm	FLOAT	mm	Cumulative rainfall last 24 hours (mm)
43	rainfall_last_15_min_in	FLOAT	in	Cumulative rainfall last 15 minutes (inches)
44	rainfall_last_15_min_mm	FLOAT	mm	Cumulative rainfall last 15 minutes (mm)
45	rainfall_last_60_min_in	FLOAT	in	Cumulative rainfall last 60 minutes (inches)
46	rainfall_last_60_min_mm	FLOAT	mm	Cumulative rainfall last 60 minutes (mm)
47	rainfall_monthly_clicks	INTEGER	-	Monthly tipping bucket cumulative counts
48	rain_storm_last_start_at	INTEGER	-	Unix timestamp of the start of the last storm
49	wind_speed_hi_last_2_min	FLOAT	mph	Maximum wind speed in the last 2 minutes
50	wind_speed_avg_last_1_min	FLOAT	mph	Average wind speed in the last minute
51	wind_speed_avg_last_2_min	FLOAT	mph	Average wind speed in the last 2 minutes
52	wind_speed_hi_last_10_min	FLOAT	mph	Maximum wind speed in the last 10 minutes
53	rainfall_last_24_hr_clicks	INTEGER	-	Bucket counts in the last 24 hours
54	wind_speed_avg_last_10_min	FLOAT	mph	Average wind speed in the last 10 minutes
55	rain_rate_hi_last_15_min_in	FLOAT	in h⁻¹	Maximum rain rate in last 15 minutes (inches)
56	rain_rate_hi_last_15_min_mm	FLOAT	mm h⁻¹	Maximum rain rate in last 15 minutes (mm)
57	rainfall_last_15_min_clicks	INTEGER	-	Bucket counts in the last 15 minutes
58	rainfall_last_60_min_clicks	INTEGER	-	Bucket counts in the last 60 minutes
59	wind_dir_scalar_avg_last_1_min	FLOAT	degrees	Scalar average wind direction (1 minute)
60	wind_dir_scalar_avg_last_2_min	FLOAT	degrees	Scalar average wind direction (2 minutes)
61	rain_rate_hi_last_15_min_clicks	INTEGER	-	Bucket counts for maximum rain rate (15 min)
62	wind_dir_at_hi_speed_last_2_min	FLOAT	degrees	Wind direction at maximum speed (last 2 min)
63	wind_dir_scalar_avg_last_10_min	FLOAT	degrees	Scalar average wind direction (10 minutes)
64	wind_dir_at_hi_speed_last_10_min	FLOAT	degrees	Wind direction at maximum speed (last 10 min)
65	bar_trend	FLOAT	inHg	Barometric trend (rate of change over 3 hours)
66	bar_offset	FLOAT	inHg	Correction factor applied to the station pressure
67	bar_absolute	FLOAT	inHg	Actual atmospheric pressure measured at the station
68	bar_sea_level	FLOAT	inHg	Corrected pressure adjusted to sea level

In addition to the 68 fields described in Table 2, which originate from the WeatherLink API JSON payload, the SQL aggregation layer that produces the export adds 7 columns required to make the export self-contained: id (surrogate primary key), station_id and station_code (station identifiers), iso_date (ISO 8601 hourly timestamp), name (human-readable station label), and the daily extremes temp_min and temp_max derived through Eqs. (5) and (6). The complete raw export therefore consists of 75 columns and is deposited in full as MeteoCoast-EC_raw.csv. Table 3 maps every one of those columns to its disposition in the processed release, and the criteria that govern the selection are described in Section 4.7.

**Table 3:** Mapping of the 75 raw columns to the 33 physical columns of the processed release. The disposition column states what happens to each group, and the basis column states the test applied: a column is discarded only if it is exactly invertible from a retained column through a fixed physical or instrumental constant, or if it encodes a quantity that is not defined at hourly resolution. Subtotals are given so that the sums can be verified directly.

Category	n	Columns	Disposition	Basis
Export identifiers	4	id, station_code, iso_date, name	Retained	Row key, station key, and hourly timestamp; required to index the series
Thermodynamic and radiative	7	temp, hum, thsw_index, solar_rad, uv_index, bar_sea_level, bar_absolute	Retained; SI equivalent added	Primary observations; not derivable from any other retained column
Daily temperature extremes	2	temp_min, temp_max	Retained and recomputed; SI equivalent added	Recomputed from the hourly series through Equations (5) and (6)
Wind statistics, WMO 10-min window	6	wind_speed_last, wind_dir_last, wind_speed_avg_last_10_min, wind_speed_hi_last_10_min, wind_dir_scalar_avg_last_10_min, wind_dir_at_hi_speed_last_10_min	Retained; speeds also SI converted	Sensor-side aggregates over the WMO reference window, defined at hourly resolution
Rainfall	4	rain_rate_last_mm, rain_rate_hi_last_15_min_mm, rainfall_daily_mm, rainfall_last_24_hr_mm	Retained	Already in SI units; span the sub-hourly to daily range
Subtotal, retained variables	23			4 identifiers + 19 measurements
Auxiliary grouping key	1	calendar_day	Added	Station-day key used by Equations (5) and (6)
SI unit conversions	9	temp_C, thsw_index_C, temp_min_C, temp_max_C, wind_speed_kmh, wind_speed_avg_10min_kmh, wind_speed_gust_10min_kmh, bar_sea_level_hPa, bar_absolute_hPa	Added	Deterministic images of retained columns under Equations (1) to (3)
Subtotal, processed release	33			4 + 19 + 1 + 9 = 33 physical columns
Imperial duplicates of SI rainfall	11	rain_storm_in, rain_rate_hi_in, rainfall_year_in, rain_rate_last_in, rainfall_daily_in, rain_storm_last_in, rainfall_monthly_in, rainfall_last_24_hr_in, rainfall_last_15_min_in, rainfall_last_60_min_in, rain_rate_hi_last_15_min_in	Discarded	Exactly invertible: 1 in = 25.4 mm
Tipping-bucket counters	11	rain_storm_clicks, rain_rate_hi_clicks, rainfall_year_clicks, rain_rate_last_clicks, rainfall_daily_clicks, rain_storm_last_clicks, rainfall_monthly_clicks, rainfall_last_24_hr_clicks, rainfall_last_15_min_clicks, rainfall_last_60_min_clicks, rain_rate_hi_last_15_min_clicks	Discarded	Exactly invertible: 1 click = 0.2 mm on the Davis rain gauge
Storm-event scoped fields	5	rain_storm_mm, rain_storm_last_mm, rain_storm_start_time, rain_storm_last_start_at, rain_storm_last_end_at	Discarded	Event-scoped and reset by the firmware; not defined at hourly resolution
Derived thermal indices	5	heat_index, thw_index, wind_chill, dew_point, wet_bulb	Discarded	Closed-form functions of retained temp, hum, and wind speed
Sub-hourly aggregates	8	rainfall_last_15_min_mm, rainfall_last_60_min_mm, wind_speed_hi_last_2_min, wind_speed_avg_last_1_min, wind_speed_avg_last_2_min, wind_dir_scalar_avg_last_1_min, wind_dir_scalar_avg_last_2_min, wind_dir_at_hi_speed_last_2_min	Discarded	1-min and 2-min windows fall below the hourly sampling cadence
Monthly and annual accumulators	2	rainfall_monthly_mm, rainfall_year_mm	Discarded	Reconstructible by summation from rainfall_daily_mm
Period-scoped peak rain rate	1	rain_rate_hi_mm	Discarded	Scoped to the firmware reporting period, superseded by the 15-min peak
Instrument and timezone metadata	5	ts, tx_id, rx_state, rain_size, tz_offset	Discarded	Non-meteorological; ts is redundant with iso_date
Pressure auxiliaries	2	bar_trend, bar_offset	Discarded	bar_trend is a finite difference of bar_sea_level; bar_offset is a calibration constant
Transmitter battery status	1	trans_battery_flag	Discarded	Instrument status rather than observation; available in the raw export
Redundant station identifier	1	station_id	Discarded	Superseded by the stable UUID station_code
Subtotal, discarded	52			11 + 11 + 5 + 5 + 8 + 2 + 1 + 5 + 2 + 1 + 1 = 52
Total, raw export	75			68 API fields + 7 SQL export fields = 75

### 3.3 Temporal completeness and missing data

No gap filling is applied, so a missing hour appears as an absent row. Completeness is the number of rows present divided by the hourly slots implied by each coverage window, counted inclusively between the first and the last hour recorded. Table 4 reports it by station, together with the longest continuous gap and the number of gaps longer than 24 hours, which is what matters for users building continuous sequences.

**Table 4:** Temporal completeness by station. Expected hourly slots are counted inclusively between the first and the last hour recorded at each station, and rows present is the number of hourly records in the processed release. Gaps are counted on the hourly grid.

Station	Coverage start	Coverage end	Expected hourly slots	Rows present	Missing hours	Completeness (%)	Longest gap (h)	Gaps > 24 h
Santa Ana	2022-01-07	2026-05-16	38,182	30,384	7,798	79.6	1,138	21
Portoviejo	2023-01-27	2026-05-16	28,925	27,453	1,472	94.9	639	5
Calderón	2025-09-08	2026-05-16	6,015	4,978	1,037	82.8	639	4
Network	2022-01-07	2026-05-16	73,122	62,815	10,307	85.9	1,138	30

Gaps have three documented causes: radio interference between the sensor suite and the datalogger, which costs one to a few hours; campus network and power outages, which affect all variables of a station at once; and sensor-specific failures, which affect a single variable. Per-variable completeness is therefore reported separately in Table 5. Two patterns there deserve comment. The reduced UV coverage at Santa Ana, mentioned only in passing in the previous version, is quantified. And the 10-minute wind quartet, the 15-minute peak rain rate, and the station-level pressure are reported by the API only from the middle of 2025 onwards, so their coverage is high at Calderón and low at the two older stations; users needing those fields for the earlier period should expect them to be absent rather than missing at random.

**Table 5:** Percentage of valid observations for each of the 19 retained measurement variables, by station and for the network. A value is counted as valid when the cell is present and numeric in the processed release, before any plausibility filter is applied.

Variable	Santa Ana (%)	Portoviejo (%)	Calderón (%)	Network (%)
temp	99.7	100.0	99.5	99.8
hum	99.7	100.0	99.5	99.8
thsw_index	99.7	100.0	99.5	99.8
solar_rad	99.7	100.0	99.5	99.8
uv_index	82.2	100.0	99.5	91.3
bar_sea_level	98.6	100.0	100.0	99.3
bar_absolute	16.6	19.8	100.0	24.6
temp_min	99.8	100.0	100.0	99.9
temp_max	99.8	100.0	100.0	99.9
wind_speed_last	99.7	100.0	99.5	99.8
wind_speed_avg_last_10_min	17.8	19.8	99.5	25.2
wind_speed_hi_last_10_min	17.8	19.8	99.5	25.2
wind_dir_last	99.7	100.0	99.5	99.8
wind_dir_scalar_avg_last_10_min	17.8	19.8	99.5	25.2
wind_dir_at_hi_speed_last_10_min	17.8	19.8	99.5	25.2
rain_rate_last_mm	99.7	100.0	99.5	99.8
rain_rate_hi_last_15_min_mm	17.8	19.8	99.5	25.2
rainfall_daily_mm	100.0	100.0	100.0	100.0
rainfall_last_24_hr_mm	100.0	100.0	100.0	100.0

## 4 Experimental Design, Materials and Methods

### 4.1 Site selection and monitoring rationale

The three MeteoCoast-EC stations were deployed in the coastal lowlands of Manabí province, Ecuador, at elevations ranging from 50 m a.s.l. at Santa Ana to 150 m a.s.l. at Calderón (Table 1) Site selection was guided by three criteria: (i) geographic complementarity within the province, ensuring a northwest-southeast transect of approximately 80 km that captures spatial variability in temperature, rainfall, and wind patterns across distinct microclimates; (ii) institutional accessibility for long-term maintenance and calibration, with each station hosted within UTM-affiliated facilities; and (iii) absence of active INAMHI stations at the target locations, establishing MeteoCoast-EC as the primary source of continuous sub-daily observations at Portoviejo and Calderón. These basins lie within a sector of the eastern Pacific where the occurrence of extreme El Niño events has substantial hydroclimatic impacts and is projected to become more frequent under continued greenhouse warming [3,4].

### 4.2 Monitoring infrastructure

All three stations are equipped with the Davis Vantage Pro 2 weather station (model 6152, Davis Instruments Corp., Hayward, CA, USA) [11]. Each unit integrates the following sensors into an Integrated Sensor Suite (ISS):

• A temperature and relative humidity sensor housed in a passive radiation shield.
• A tipping-bucket rain gauge with 0.2 mm resolution per tip.
• An anemometer for wind speed (resolution: 1 km h⁻¹) and wind direction (resolution: 1°).
• A silicon photodiode solar radiation pyranometer.
• A UV sensor calibrated to the standard WHO UV Index scale (0–11+).

The wireless ISS transmits readings to a WeatherLink Live (model 6100) datalogger every 2.5 seconds. The datalogger connects via Ethernet to the UTM campus network and uploads the readings to the WeatherLink cloud service, from which the UTM acquisition service retrieves them through the WeatherLink v2 cloud API (Section 4.3). The Davis Vantage Pro 2 meets WMO accuracy guidelines for automatic weather stations, with temperature accuracy of ±0.5°C, rainfall resolution of 0.2 mm, and wind speed accuracy of ±2 km h⁻¹ [12].

**Calibration, maintenance, and inspection.** The units were commissioned on 7 January 2022 at Santa Ana, 27 January 2023 at Portoviejo, and 31 August 2025 at Calderón, whose first valid hourly record dates from 8 September 2025, each with a factory calibration certificate and no subsequent field recalibration against a WMO-traceable reference. Preventive maintenance is performed every six months by UTM technical staff, and covers the rain gauge funnel, the radiation shield, the anemometer and vane, the pyranometer dome, and the radio and Ethernet links. The log of interventions is deposited as station_maintenance_log.csv so that a suspected discontinuity can be aligned with a documented visit.

### 4.3 Data acquisition and storage

The WeatherLink v2 cloud API exposes the current record of each station as a JSON document in which the readings are grouped by sensor block. A JavaScript-based polling service queries the current endpoint of every station every 60 seconds and stores three of those blocks, the console diagnostics, the barometer, and the integrated sensor suite, as JSONB documents in a PostgreSQL (v14) relational database hosted on a UTM server, together with the station UUID (station_code), the numeric identifier (station_id), and the timestamp reported by the API. That timestamp is stored in UTC and written to the export in Ecuador local civil time with the offset appended (iso_date, ending in -0500); calendar_day and the daily temperature extremes are defined on the local civil day (UTC-5), not on the UTC day. The database schema and the polling service are deposited as schema.sql and etl_weatherlink_poller.js, so that the acquisition layer can be reconstructed independently of this article. The service reads the API key and secret from the environment, so no credential is contained in the deposited code.

### 4.4 Data processing and export

The MeteoCoast-EC CSV file was generated through a SQL query applying the following sequential transformations to the raw one-minute records:

• Temporal aggregation: minute-level readings are grouped by station and calendar hour, and the instantaneous value closest to the top of each hour is retained for all variables. The result is an hourly point sample and not an hourly mean; the justification and the consequences are given at the end of this section.
• Daily statistics: temp_min and temp_max are the daily extremes of the valid hourly temperature observations of each station-day, as defined by Equations (5) and (6); build_release.py computes them on the hourly series of the processed release. In the raw export these two columns are the export-layer daily extremes computed over every record of the station-day, so they can differ from the processed values.
• Deduplication: where two or more records share station_code and iso_date, the record with the lowest id, that is the earliest ingestion, is retained; build_release.py sorts by id before removing duplicates, so the rule is deterministic. These duplicates arise from retried or re-ingested polls. Of the 1,171,323 raw records, 33,614 are duplicates under that key: 27,144 are exact copies and 6,470, in 3,495 station-hours, carry differing sensor values, the latter produced by two overlapping ingestion passes that recorded a reading under different unit conventions. 1,137,709 distinct records enter the hourly aggregation.
• Export: the resulting table is exported as a UTF-8 CSV with comma delimiter and a single header row; no imputation or gap-filling is applied, so missing hourly observations appear as absent rows.

Retaining a point sample rather than an hourly mean is deliberate. The payload already carries sensor-side aggregates over the WMO 10-minute window, so averaging them again would compound two levels of smoothing under an undocumented weighting. The polling interval is nominally 60 s but not strictly regular, and an unweighted mean over an irregularly sampled hour is biased toward the densely sampled sub-intervals whereas a point sample is not. Several retained variables are also not meaningfully averageable: the rainfall accumulators are running totals and wind_dir_last is circular.

The cost is that point samples carry higher variance and heavier tails than true hourly means, most noticeably for wind speed during convective activity. For users who need means, hourly_mean_aggregation.sql is deposited. The export and aggregation layers themselves are deposited as export_raw_75col.sql and build_release.py, the latter applying the hourly aggregation described above.

The retained observation lies close to the hour boundary. Its offset from the top of the hour has a median of 0 s at Santa Ana and Portoviejo and 30 s at Calderon, a 95th percentile between 45 and 57 s, and is within 60 s in 99.5 percent of hours; in fewer than 0.3 percent of hours, where the only available record lies far from the boundary, the offset can reach up to 59 min. Ties, which occur in 11.5 percent of hours, are broken deterministically by the lowest id, and no maximum offset is imposed, so every hour with at least one observation is represented by its closest available record. The selection is applied per variable, so a row may combine values retained from more than one sub-hourly record.

### 4.5 Unit conventions and conversion factors

Sensor readings are stored using the native encoding delivered by the Davis WeatherLink API. Therefore, before any analytical step or comparison with other datasets, the following conversions should be applied.

Air temperature, dew point, wet bulb, and the heat, wind-chill, THW, and THSW indices are recorded in degrees Fahrenheit (°F). Conversion to degrees Celsius (°C) is given by Equation (1):

(1) $$T_{C}=\left(T_{F}-32\right)\times 5/9$$

where *T*_°C_ is the temperature expressed in degrees Celsius and *T*_°F_ is the temperature in degrees Fahrenheit, as delivered by the sensor.

Barometric pressure variables (bar_absolute, bar_sea_level, bar_offset, bar_trend) are reported by the Davis WeatherLink API in inches of mercury (inHg), the native unit of the Davis Vantage Pro 2 sensor; values therefore range around 29.9 inHg (1,013 hPa) for sea-level pressure. Conversion to hectopascals (hPa) is obtained by Equation (2):

(2) $$p_{\text{hpa}}=\text{bar}\_\text{sea}\_\text{level}\times 33.8639$$

where *p*_hPa_ is the barometric pressure adjusted to sea level expressed in hectopascals, bar_sea_level is the raw value reported by the WeatherLink API in inches of mercury, and 33.8639 is the unit-conversion factor between inHg and hPa.

Wind speed is recorded in miles per hour (mph). Conversion to kilometers per hour (km h⁻¹) is given by Equation (3):

(3) $$V_{\text{km}\;h^{-1}}=V_{\text{mph}}\times 1.609344$$

where *v*_km h⁻¹_ is wind speed in kilometers per hour and *v*_mph_ is the raw value in miles per hour as reported for wind_speed_last and related fields.

The THSW index is also expressed in°F. Values above 29,000 are sentinel codes emitted by the Davis algorithm when solar radiation or wind inputs are unavailable, typically at night, and these records should be excluded from thermal comfort analyses. A filter for valid THSW values is defined in Equation (4):

(4) $$\text{THS}W_{\text{valid}}=\geq \left\{\times \in \text{THSW}:\times <29,000\right\}$$

where THSW_valid_ denotes the subset of THSW index observations retained for analysis once sentinel values are removed.

**Unit conventions across the reporting period.** The barometric field is not delivered in a single unit throughout the record. Most of it arrives in inches of mercury, a smaller part in millimetres of mercury, and a further part with the inHg value scaled by one thousand. The conversion of Equation (2) is therefore applied per record according to the convention detected from the magnitude of the value, and the native column is deposited exactly as delivered. A subset of records concentrated in 2023 and 2024 carries the field in a scale that could not be identified, and for those the SI column is left empty rather than converted under a guess; the same records report the THSW index in degrees Celsius rather than Fahrenheit, which the conversion also detects. Table 6 reports how many observations each rule removes from the descriptive subset. The convention is identified from the magnitude of the value: 27 to 32 for inches of mercury, 700 to 800 for millimetres of mercury, and above 1,000 for the inHg value scaled by one thousand; values outside these ranges cannot be resolved and leave the SI column empty. In the processed release these amount to 24,569 records resolved (15,450 in inHg, 7,639 in mmHg and 1,480 scaled), 37,808 in an unidentified scale left unconverted, and 438 genuinely missing. The unidentified scale is concentrated at Portoviejo (22,014 records) and Santa Ana (15,794) in 2023 and 2024, whereas the record from 2025 onwards is uniformly in inHg. The large exclusion of sea-level pressure from the descriptive subset in Table 6 is therefore dominated by unresolved unit conventions rather than by missing sensor readings.

**Table 6:** Sentinel rule and plausibility interval applied to each retained measurement variable by the descriptive cleaning pipeline, with the number of observations removed. The intervals are applied in the native units of the raw export; the SI equivalents are given for reference. For the barometric field and the THSW index the rule is evaluated on the SI column, which resolves the unit conventions described in Section 4.5. These rules are not applied to the released files. Section 4.6 distinguishes limits taken from the manufacturer operating range from study-specific plausibility limits.

Variable	Sentinel rule	Plausibility interval (native)	Equivalent (SI)	Observations removed
temp	-	50 to 110°F	10.0 to 43.3°C	21
hum	-	0 to 100 %	0 to 100 %	0
thsw_index	x < 29,000°F, Eq. (4)	50 to 130°F	10.0 to 54.4°C	11,045
solar_rad	-	0 to 1,600 W m⁻²	0 to 1,600 W m⁻²	0
uv_index	-	0 to 16	0 to 16	0
bar_sea_level	-	27 to 32 inHg	914.3 to 1,083.6 hPa	37,808
bar_absolute	-	26 to 31 inHg	880.5 to 1,049.8 hPa	0
temp_min	-	derived from temp	derived from temp	recomputed
temp_max	-	derived from temp	derived from temp	recomputed
wind_speed_last	-	0 to 200 mph	0 to 321.9 km h⁻¹	0
wind_speed_avg_last_10_min	-	0 to 200 mph	0 to 321.9 km h⁻¹	0
wind_speed_hi_last_10_min	-	0 to 200 mph	0 to 321.9 km h⁻¹	0
wind_dir_last	-	0 to 360°	0 to 360°	0
wind_dir_scalar_avg_last_10_min	-	0 to 360°	0 to 360°	0
wind_dir_at_hi_speed_last_10_min	-	0 to 360°	0 to 360°	0
rain_rate_last_mm	-	0 to 500 mm h⁻¹	0 to 500 mm h⁻¹	0
rain_rate_hi_last_15_min_mm	-	0 to 500 mm h⁻¹	0 to 500 mm h⁻¹	0
rainfall_daily_mm	-	0 to 500 mm	0 to 500 mm	0
rainfall_last_24_hr_mm	-	0 to 500 mm	0 to 500 mm	0

Finally, the variables temp_min and temp_max are the true daily extremes of the hourly temperature series, obtained by grouping the valid hourly observations on station and calendar day. Given the set of valid hourly temperature observations T(s,d,h) recorded at station s on day d, they are defined by Equations (5) and (6):

(5) temp_min(s,d)=minoverhinH(s,d)ofT(s,d,h)

(6) temp_max(s,d)=maxoverhinH(s,d)ofT(s,d,h)

where H(s,d) is the set of hours with a valid temperature observation at station s on day d. Two properties matter for reuse. The daily value is broadcast to all hourly rows of the same station-day, so users must collapse on calendar_day before any daily statistic. And the extremes are taken over hourly point samples rather than over the minute-level record, so they are conservative. The original field names are kept for traceability, and the aliases temp_daily_min and temp_daily_max are registered in the data dictionary.

### 4.6 Quality control and cleaning applied for descriptive characterization

The two released files are not quality-controlled beyond deduplication. They carry the native sensor values, including sentinel codes and readings outside a physically plausible interval, and no value is deleted, capped, flagged, or imputed. Four transformations, and only these, produce the processed release from the raw export: the hourly point sampling of Section 4.4, deduplication on station_code and iso_date, the recomputation of the daily temperature extremes, and the 9 SI conversions. None of them removes an observation on quality grounds.

The descriptive statistics of Table 7 and the two figures of Section 4.7 are computed on a cleaned subset produced by a separate pipeline whose output is not deposited. It applies the THSW sentinel rule of Equation (4), then a plausibility interval to each numeric variable in the native units of the raw export, aligned with the instrument specification and with WMO guidance on quality control [13], and then recomputes the daily extremes on the surviving observations. Table 6 gives the rule, the interval, and the number of observations removed for every variable, so the descriptive results can be reproduced exactly. The pipeline is deposited as cleaning_pipeline.R and the figure code as figures.R. Table 6 combines two kinds of bound. Limits taken directly from the manufacturer operating range of the Davis Vantage Pro 2 (the 0 to 100 percent humidity range, the 0 to 360 degree direction range, the wind-speed ceiling of 200 mph, the solar-radiation ceiling of 1,600 W m-2 and the barometric ranges) mark instrument errors. Study-specific plausibility limits chosen by the authors for this coastal setting (the 50 to 110 degree F air-temperature window, the 50 to 130 degree F THSW window and the 500 mm h-1 rainfall ceiling) mark values judged climatologically implausible for the descriptive characterisation. A value outside a study-specific window is not necessarily an instrument error, and both criteria are identified as such in the deposited data dictionary.

**Table 7:** Description and descriptive statistics of the 23 variables retained in the processed MeteoCoast-EC release, computed on the cleaned subset of Section 4.6. The valid-observation count n is reported for each variable; counts by station are deposited in table7_valid_counts_by_station.csv. For wind_speed_last and rain_rate_last_mm the statistics use the reliable era only (from the onset of the 10-minute statistics).

N°	Variable	n (valid)	Min	Max	Mean	Std	Unit	Notes
1	id	-	-	-	-	-	-	Primary key (row identifier)
2	station_code	-	-	-	-	-	-	Station UUID code
3	iso_date	-	-	-	-	-	UTC-5	ISO 8601 hourly timestamp
4	name	-	-	-	-	-	-	Station display name
5	temp	62,691	15.00	41.00	25.71	3.16	°C	Convert with Eq. (1) if°F is required
6	hum	62,711	0.00	99.00	76.71	10.11	%	Relative humidity; required for Penman-Monteith ET₀ and VPD
7	thsw_index	51,665	15.56	53.83	26.81	5.13	°C	Composite T+H+sun+wind comfort index; filter via Eq. (4)
8	solar_rad	62,710	0.00	1,440.00	74.43	164.92	W m⁻²	0 during night hours
9	uv_index	57,375	0.00	11.70	0.24	1.05	index	0 during night hours; WHO scale 0–11+
10	bar_sea_level	24,569	1,005.05	1,018.39	1,011.91	1.98	hPa	Sea-level pressure; convert via Eq. (2) if inHg is required
11	bar_absolute	15,450	984.66	1,011.14	1,000.27	6.65	hPa	Station-level pressure; required for boundary-layer studies
12	temp_min	2,685	15.00	29.28	22.49	2.69	°C	Daily minimum recomputed via Eq. (5); n per station-day
13	temp_max	2,685	19.00	41.00	29.46	2.83	°C	Daily maximum recomputed via Eq. (6); n per station-day
14	wind_speed_last	15,808	0.00	35.41	2.76	3.69	km h⁻¹	Instantaneous; convert via Eq. (3) from mph; reliable era only
15	wind_dir_last	62,711	0.00	360.00	74.63	90.42	°	Instantaneous wind direction (0°=N, clockwise)
16	wind_speed_avg_last_10_min	15,808	0.00	15.58	2.50	2.32	km h⁻¹	WMO standard 10-min mean wind speed
17	wind_speed_hi_last_10_min	15,807	0.00	114.26	7.40	5.53	km h⁻¹	WMO standard 10-min peak gust
18	wind_dir_scalar_avg_last_10_min	15,805	0.00	360.00	143.83	104.37	°	WMO 10-min scalar-mean wind direction
19	wind_dir_at_hi_speed_last_10_min	15,807	0.00	360.00	149.75	107.25	°	Wind direction at 10-min peak gust
20	rain_rate_last_mm	15,809	0.00	90.00	0.70	6.19	mm h⁻¹	Instantaneous rain rate at observation time; reliable era only
21	rain_rate_hi_last_15_min_mm	15,809	0.00	86.00	0.51	4.26	mm h⁻¹	15-min peak rain rate; required for IDF curves
22	rainfall_daily_mm	62,815	0.00	47.50	0.25	2.10	mm	Daily cumulative; resets at midnight
23	rainfall_last_24_hr_mm	62,815	0.00	94.60	0.46	3.54	mm	Rolling 24-hour cumulative rainfall

The separation matters for reuse: a user studying thermal extremes needs the unfiltered record, while a user computing monthly climatologies needs the filtered subset. Publishing the files unfiltered and the filter as code lets both proceed from the same deposit.

### 4.7 Processed dataset and descriptive statistics

The raw export contains 75 columns, 68 from the API payload plus 7 added by the export query. The processed release retains 23 and discards 52, under four principles applied uniformly: (P1) independence of information; (P2) compatibility with the hourly sampling resolution; (P3) alignment with WMO observational standards [9]; and (P4) sufficiency for the use cases of Section 2. Following the reviewer's suggestion, the complete raw export is now deposited, so the selection is a convenience rather than a restriction: dew point, wet bulb, the heat and wind-chill indices, storm-event fields, tipping-bucket counts, the 1-min and 2-min wind statistics, and the battery flag can be read from MeteoCoast-EC_raw.csv and joined on station_code and iso_date.

The principles are tested column by column, and the test is recorded for every discard in Table 3. A column is discarded only if it is exactly invertible from a retained column through a fixed constant, as with the imperial duplicates under 1 in = 25.4 mm and the counters under 1 click = 0.2 mm, or if it encodes a quantity not defined at hourly resolution, as with the storm-scoped fields and the 1-min and 2-min wind statistics. Any column failing both tests is retained, which is why the 10-minute wind quartet and the 15-minute peak rain rate are kept.

The 52 discarded columns are 11 imperial duplicates of SI rainfall variables, 11 tipping-bucket counters, 5 storm-event scoped fields, 5 derived thermal indices (heat_index, thw_index, wind_chill, dew_point, wet_bulb), 8 sub-hourly aggregates at 1-min and 2-min windows together with the 15-min and 60-min rolling rainfall totals, 2 monthly and annual accumulators, 1 period-scoped peak rain rate (rain_rate_hi_mm), 5 instrument and timezone metadata fields, 2 pressure auxiliaries, the transmitter battery flag, and the redundant numeric station_id. The sum is stated explicitly because the previous version did not add up: 11 + 11 + 5 + 5 + 8 + 2 + 1 + 5 + 2 + 1 + 1 = 52.

The resulting 23 variables comprise: 4 export-level identifiers (id, station_code, iso_date, name); 7 thermodynamic and radiative observations (temp, hum, thsw_index, solar_rad, uv_index, bar_sea_level, bar_absolute); 2 derived daily temperature extremes (temp_min, temp_max) recomputed through Equations (5) and (6); 6 WMO-aligned wind statistics combining the instantaneous reading with the 10-min average and gust quartet (wind_speed_last, wind_dir_last, wind_speed_avg_last_10_min, wind_speed_hi_last_10_min, wind_dir_scalar_avg_last_10_min, wind_dir_at_hi_speed_last_10_min); 4 rainfall variables capturing the full range of relevant temporal scales from sub-hourly peaks to rolling daily accumulation (rain_rate_last_mm, rain_rate_hi_last_15_min_mm, rainfall_daily_mm, rainfall_last_24_hr_mm).

The 23 retained variables are materialized in the distributed CSV as 33 physical columns: 4 export-level identifiers, 1 auxiliary calendar_day, 19 retained variables in their native Davis units, plus 9 SI-converted equivalents (temp_C, thsw_index_C, temp_min_C, temp_max_C, wind_speed_kmh, wind_speed_avg_10min_kmh, wind_speed_gust_10min_kmh, bar_sea_level_hPa, bar_absolute_hPa) derived deterministically from the native columns through Equations (1)–(3) and included as a convenience for downstream analysis without inflating the count of analytically distinct observations.

Table 7 describes the retained variables and reports descriptive statistics computed over the cleaned subset defined in Section 4.6, not over the released files. Every statistic is computed on the cleaned subset after sentinel removal and plausibility filtering, and the valid-observation count n is reported for each variable, with counts by station deposited in table7_valid_counts_by_station.csv. For the instantaneous wind speed (wind_speed_last) and the instantaneous rain rate (rain_rate_last_mm), whose earlier values are affected by the field-mapping problem of Section 5, the statistics are computed on the reliable era only.

**Fig. 2:**
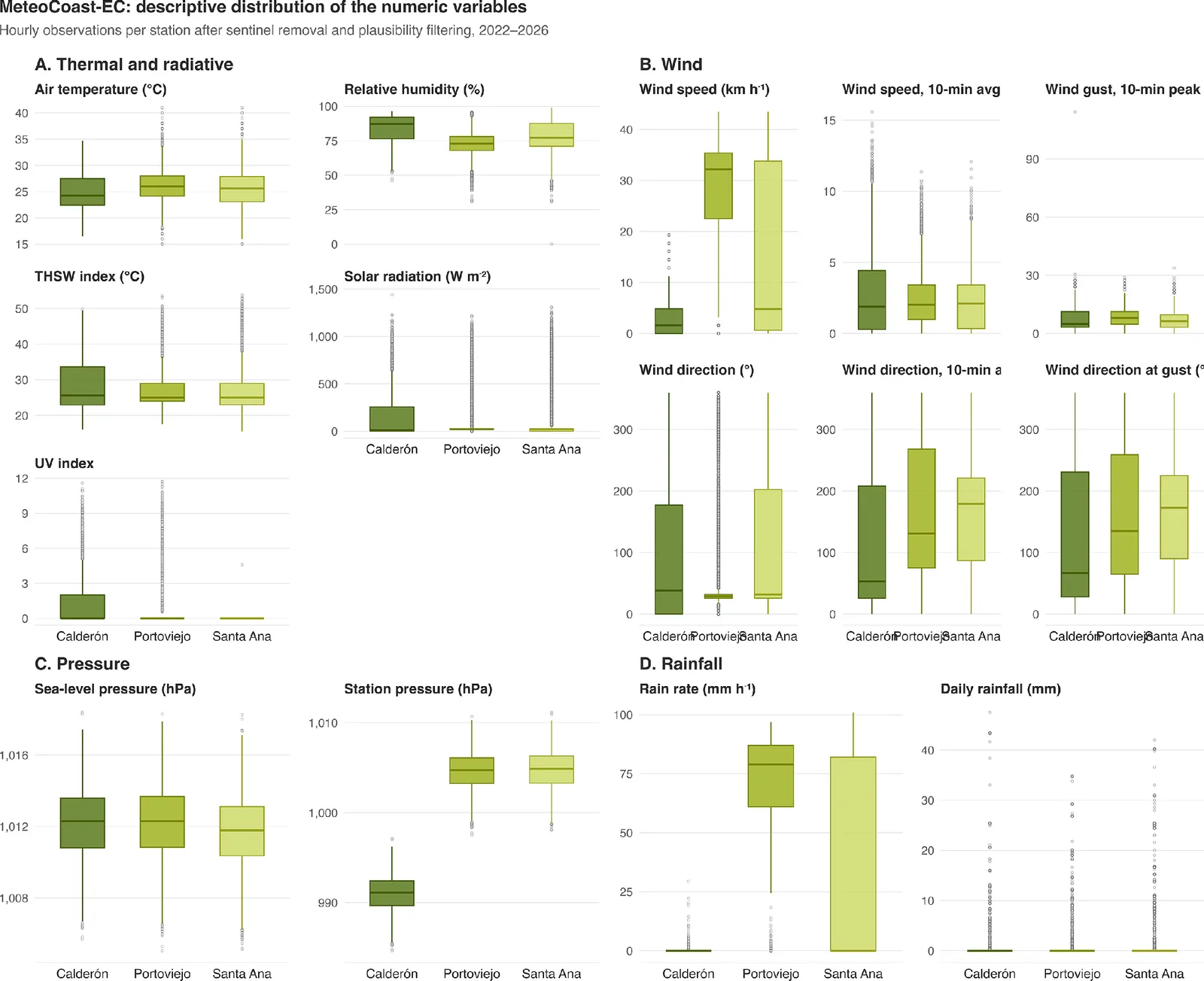
Descriptive distribution of the numeric variables of the cleaned subset across the three MeteoCoast-EC stations (Calderón, Portoviejo, Santa Ana), organised into four thematic panels: (A) thermal and radiative, with air temperature, relative humidity, the THSW index, solar radiation and the UV index; (B) wind, with the instantaneous speed and direction and the WMO 10-minute statistics; (C) pressure, at sea level and at station level; and (D) rainfall, with the instantaneous rate and the daily accumulation. Each variable carries a free vertical scale. Boxplots summarize the hourly observations retained after sentinel removal and plausibility filtering, 2022 to 2026; whiskers extend to 1.5 times the interquartile range and circles denote retained extremes. The figure is supplied at 600 dpi and each panel is also deposited as a separate file at the same resolution. The three stations cover different periods (Santa Ana from January 2022, Portoviejo from January 2023, Calderon from September 2025), so differences among the station boxplots reflect unequal temporal coverage as well as spatial climate; a like-for-like comparison over the concurrent window, September 2025 to May 2026, is given in the text.

**Fig. 3:**
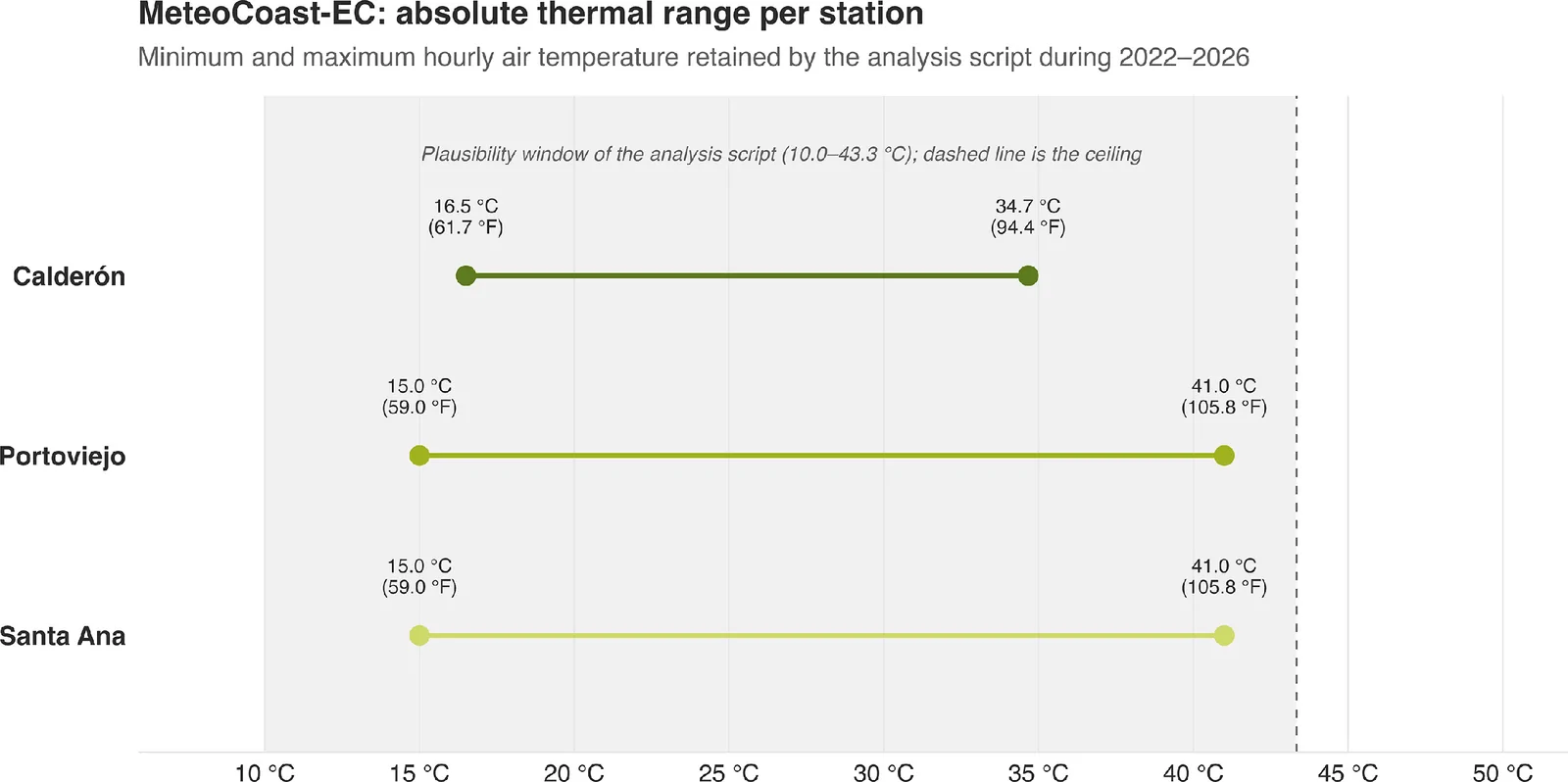
Absolute thermal range per station during 2022 to 2026, computed on the cleaned subset of Section 4.6. Endpoints indicate the minimum and maximum hourly air temperature retained by the plausibility filter, in degrees Celsius and Fahrenheit. The shaded band is the filter window itself, 50 to 110°F, that is 10.0 to 43.3°C, and the dashed line marks its ceiling. No station maximum reaches the ceiling, so no endpoint is censored. The window is a property of the analysis script and not of the released files. The endpoints are the extrema of the cleaned descriptive subset, not of the released files, which retain the values above the ceiling; the raw observed maxima can be obtained from MeteoCoast-EC_raw.csv or with validation_checks.R.

Two descriptive figures characterize the cleaned subset. Fig. 2 reports the distribution of the numeric variables by station, organised into four thematic panels, and Fig. 3 reports the absolute thermal envelope per station.

Fig. 2 presents the variables of the cleaned subset that admit a meaningful distributional summary, grouped by family. Panel A collects the thermal and radiative variables. Air temperature is similar across the three sites, with medians between 24 and 26°C and interquartile ranges of roughly 22 to 28°C; Calderón shows the lowest median, consistent with its 150 m a.s.l. elevation. Relative humidity is uniformly high and separates the sites, with a median near 87% at Calderón against 77% at Santa Ana and 73% at Portoviejo, which confirms the persistent high-moisture regime that drives the agroclimatic relevance of the dataset. The THSW comfort index is centred around 25 to 26°C with upper tails beyond 50°C during midday hours of high solar load and low ventilation. Solar radiation and the UV index are dominated by low night-time values at all three stations, with Calderón showing the widest interquartile range and Santa Ana the reduced UV coverage quantified in Table 5.

Panel C carries the clearest spatial signal. Sea-level pressure clusters between 1,008 and 1,016 hPa at all three stations with medians near 1,012 hPa, which validates the altitude reduction of Equation (2), while station pressure separates the network into two altitude regimes, with Calderón near 991 hPa against about 1,005 hPa at Portoviejo and Santa Ana. Panels B and D require a caveat. The WMO 10-minute wind statistics, available from the middle of 2025 onwards, are internally consistent, with mean speeds below 5 km h⁻¹ and gusts rarely above 20 km h⁻¹. The instantaneous wind speed and the instantaneous rain rate recorded before that date at Portoviejo and Santa Ana are not: they are inconsistent with the concurrent 10-minute means and with the daily accumulations of panel D, which points to a field-mapping problem in the earlier acquisition software rather than to a meteorological signal. Those two fields should not be used for the earlier period, and the limitation is recorded in Section 5.

Restricted to the concurrent window of September 2025 to May 2026, over which all three stations report, the spatial pattern holds without the confound of unequal coverage: median air temperature is lowest at Calderon (24.2 degrees C) and near 24.8 degrees C at the two lower sites, median relative humidity is highest at Calderon (87 percent) against 85 percent at Santa Ana and 83 percent at Portoviejo, the station-pressure stratification persists (about 991 hPa at Calderon against about 1,005 hPa at the two lower sites), and the WMO 10-minute mean wind speed stays below 3 km h-1 at all three. Ultraviolet radiation is the exception: the Santa Ana UV channel returns no valid observation in this window, so its UV distribution is not comparable.

Fig. 3 summarizes the absolute thermal envelope of each station. The shaded band is the plausibility window of Table 6, 10.0 to 43.3°C, applied by the cleaning pipeline and not by the export that produces the released files. Calderón shows the narrowest envelope, 16.5 to 34.7°C, consistent with its elevation, while Portoviejo and Santa Ana both span 15.0 to 41.0°C. No station maximum reaches the ceiling, so no endpoint is censored. The filter does truncate the upper tail, since 20 hourly observations lie above the ceiling and are removed for the descriptive characterization while remaining in both released files unaltered. Users studying temperature extremes should recompute the filter with a higher ceiling on the raw file and screen the upper tail against the concurrent solar radiation and 10-minute mean wind speed, discarding as radiation-shield artefacts those maxima that coincide with high radiation and near-calm wind; that diagnostic is implemented in validation_checks.R.

The combined evidence from Fig. 2, Fig. 3 supports the validity of the descriptive cleaning procedure: extreme but physically credible values are preserved, including rainfall peaks, gust extremes, and the upper THSW tail; the cross-station consistency expected on physical grounds is recovered, including sea-level pressure homogeneity, station pressure stratification by altitude, and similar temperature regimes; and the daily extremes are now the true daily minima and maxima defined by Equations (5) and (6) rather than the quantity described in the previous version.

### 4.8 Internal consistency of the network

No co-located reference station is available at Portoviejo or Calderón, which is the gap that motivates the dataset, so a direct validation against a national reference is not possible. We report an internal consistency check instead, implemented in the deposited validation_checks.R.

Sea-level pressure should be spatially coherent over distances of tens of kilometres, so the three series are compared pairwise over the 4,977 hours in which all three stations report. The mean pairwise correlation is 0.95 and the mean absolute bias is 0.30 hPa, with station means agreeing to better than 0.5 hPa. That is the agreement expected of correctly operating barometers at these separations, and the procedure follows published guidance for automatic weather station data [14]. The check establishes that the three barometers agree with one another; it does not substitute for traceable absolute calibration.

## Limitations

MeteoCoast-EC presents six main limitations. First, temporal coverage is unequal: Calderón joined in August 2025 and provides about nine months of records, against about four years for Santa Ana and three for Portoviejo, so balanced comparisons should be restricted to the concurrent window of August 2025 to May 2026. Second, variables are stored in the native units of the WeatherLink API and a subset of records carries non-numeric sentinel codes, so users must apply the conversions of Section 4.5 or read the SI columns of the processed release. Third, neither released file is quality-controlled beyond deduplication, so sensor malfunctions, calibration drift, radiation-shield heating, and transmission errors may introduce unflagged outliers; the pipeline of Section 4.6 is provided as code and its output is not the released file, so users requiring controlled data must run it or apply their own checks following WMO guidance [13]. Fourth, the hourly values are point samples rather than hourly means, so they carry higher variance and heavier tails; hourly_mean_aggregation.sql is deposited for users who need means. Fifth, each station operates a single unit without a redundant reference instrument and no field recalibration against a traceable reference has been performed, so the check of Section 4.8 rests on the internal coherence of the network; spatial generalization additionally requires geostatistical interpolation not included here [15]. Sixth, the instantaneous wind speed and the instantaneous rain rate recorded before the middle of 2025 at Portoviejo and Santa Ana are inconsistent with the concurrent 10-minute wind statistics and with the daily rainfall accumulations, which indicates a field-mapping problem in the earlier acquisition software; both fields are released unaltered but should not be used for that period, where the 10-minute statistics and the daily accumulations should be preferred; the exact affected intervals are listed in the deposited quality_flags.csv.

## Data Availability

All data and code required to reproduce every table and figure of this article are deposited in Mendeley Data under https://www.doi.org/10.17632/bpsh7jtyd2.5 under CC BY 4.0, following the FAIR principles [12]. Every file named in this article is contained in the version of that record cited here, organised in four folders, data, docs, code and figures; no item is distributed as supplementary material to the article, and no API credential is included, since the acquisition service reads its key and secret from the environment. The deposit contains the two data files, MeteoCoast-EC_raw.csv and MeteoCoast-EC_processed.csv; the documentation files MeteoCoast-EC_data_dictionary.csv and MeteoCoast-EC_data_dictionary.json, station_metadata.csv, station_maintenance_log.csv, stations.geojson, quality_flags.csv, and README.md; and the code: schema.sql and etl_weatherlink_poller.js for acquisition, export_raw_75col.sql and build_release.py for the export and aggregation layers, hourly_mean_aggregation.sql, cleaning_pipeline.R, compute_tokens.py, figures.R, and validation_checks.R.

Table 1, Table 4 to 7 are regenerated by cleaning_pipeline.R over MeteoCoast-EC_processed.csv, Table 3 from the header of MeteoCoast-EC_raw.csv, Figure 1 from stations.geojson with figures.R, and Fig. 2, Fig. 3 by figures.R. For every column of both files the data dictionary records the name, any alias, the type, the unit, the measurement frequency, the valid range used by the pipeline, the missing-value code, whether the column is measured, derived, or converted, and the governing equation where one applies.

## Ethics Statement

The data described in this article were collected by automatic weather stations operated by the Universidad Técnica de Manabí for scientific, educational, and public service purposes. No human subjects, animal subjects, or personally identifiable information are involved. The dataset is released under the Creative Commons Attribution 4.0 International License (CC BY 4.0), which permits unrestricted use, distribution, and reproduction in any medium, provided that the original work is properly cited.

## CRediT authorship contribution statement

**Jorge Parraga-Alava:** Conceptualization, Software, Data curation, Investigation, Validation, Visualization, Project administration, Writing – original draft, Writing – review & editing. **Edison Solorzano:** Software, Data curation, Visualization, Validation, Investigation, Writing – review & editing. **Emilio Jarre-Castro:** Conceptualization, Methodology, Investigation, Writing – review & editing. **Jose Zambrano:** Software, Visualization, Writing – review & editing. **Ezequiel Zamora-Ledezma:** Conceptualization, Methodology, Resources, Investigation, Project administration, Supervision, Writing – original draft, Writing – review & editing.

## References

[1] Instituto Nacional de Meteorología e Hidrología (INAMHI). Red de Estaciones Meteorológicas del Ecuador. Quito, Ecuador: INAMHI; 2023. Available from: https://www.inamhi.gob.ec.

[2] Funk C.C., Peterson P.J., Landsfeld M.F., Pedreros D.H., Verdin J.P., Shukla S. (2015). The climate hazards infrared precipitation with stations – a new environmental record for monitoring extremes. Sci. Data.

[3] Thielen D.R., Ramoni-Perazzi P., Zamora-Ledezma E., Puche M.L., Marquez M., Quintero J.I. (2023). Effect of extreme El Niño events on the precipitation of Ecuador. Nat. Hazards Earth Syst. Sci..

[4] Cai W., Borlace S., Lengaigne M., van Rensch P., Collins M., Vecchi G. (2014). Increasing frequency of extreme El Niño events due to greenhouse warming. Nat. Clim. Chang..

[5] Mantuano García D.L., Luque Vera J.C. (2025). Sector agropecuario y su aporte en el crecimiento económico de la provincia de Manabí – Ecuador. Rev. Cienc. Soc. Econ..

[6] Mora-Felix Z.D., Monjardin-Armenta S.A., Rangel-Peraza J.G., Renteria-Guevara S.A., Sanhouse-Garcia A.J., Bustos-Terrones Y. (2025). 92-year meteorological datasets from 50 meteorological stations in Sinaloa, Mexico with daily, monthly, and yearly resolutions. Data Br..

[7] Zubair M., Mahee M.N.I., Reza K.M., Salim M.S., Ahmed N. (2024). Climate data dynamics: a high-volume real world structured weather dataset. Data Br..

[8] Moreira G.A., Carbone S., Guerrero-Rascado J.L., Andrade I.S., Cacheffo A., Vélez-Pereira A.M. (2025). Evidence of the consequences of the prolonged fire season on air quality and public health from 2024 São Paulo (Brazil) data. Sci. Rep..

[9] World Meteorological Organization (WMO). Guide to Instruments and Methods of Observation (WMO-No. 8). Geneva: WMO; 2018. Available from: https://library.wmo.int/records/item/41650-guide-to-instruments-and-methods-of-observation.

[10] World Meteorological Organization (WMO) (2013). Manual on the Global Observing System. Volume I – Global aspects (WMO-No. 544).

[11] Davis Instruments Corp. WeatherLink Live Installation and Operation Manual. Hayward, CA, USA: Davis Instruments; 2021. Available from: https://www.davisinstruments.com/products/weatherlinklivewifi/.

[12] World Meteorological Organization (WMO) (2013).

[13] Zahumenský I. (2004).

[14] Estévez J., Gavilán P., Giráldez J.V. (2011). Guidelines on validation procedures for meteorological data from automatic weather stations. J. Hydrol..

[15] Zahumenský I. Guidelines on Quality Control Procedures for Data from Automatic Weather Stations. Geneva: World Meteorological Organization, Commission for Instruments and Methods of Observation; 2004. Available from: https://library.wmo.int/records/item/51162-guidelines-on-quality-control-procedures-for-data-from-automatic-weather-stations.

